# A Novel Peptide-Binding Motifs Inference Approach to Understand Deoxynivalenol Molecular Toxicity

**DOI:** 10.3390/toxins7061989

**Published:** 2015-06-02

**Authors:** Yousef I. Hassan, Christena Watts, Xiu-Zhen Li, Ting Zhou

**Affiliations:** Guelph Food Research Centre, Agriculture and Agri-Food Canada (AAFC), Guelph, ON N1G 5C9, Canada; E-Mails: yousef.hassan@agr.gc.ca (Y.I.H.); christina.watts@agr.gc.ca (C.W.); xiu-zhen.li@agr.gc.ca (X.-Z.L.)

**Keywords:** deoxynivalenol, toxicity, modification, enzyme, computational biology

## Abstract

Deoxynivalenol (DON) is a type B trichothecene mycotoxin that is commonly detected in cereals and grains world-wide. The low-tolerated levels of this mycotoxin, especially in mono-gastric animals, reflect its bio-potency. The toxicity of DON is conventionally attributed to its ability to inhibit ribosomal protein biosynthesis, but recent advances in molecular tools have elucidated novel mechanisms that further explain DON’s toxicological profile, complementing the diverse symptoms associated with its exposure. This article summarizes the recent findings related to novel mechanisms of DON toxicity as well as how structural modifications to DON alter its potency. In addition, it explores feasible ways of expanding our understating of DON-cellular targets and their roles in DON toxicity, clearance, and detoxification through the utilization of computational biology approaches.

## 1. Introduction

Type B trichothecene mycotoxins are common contaminants of grains and cereals around the world. The presence of these fungal secondary-metabolites raises public-health concerns at both the agriculture and food industry levels. At the top of the trichothecenes list comes deoxynivalenol (DON), the most widely detected mycotoxin in feed and food products in both developed and developing countries. In fact, a recent study showed that DON was by far the leading contaminant in feed ingredients including corn, wheat bran, soybean meal, and dried distillers grains with solubles (DDGS) used in the Chinese market [1]. Similarly, a three-year survey of North American grains found that 79% of corn and 76% of wheat/wheat bran samples were contaminated by DON [2]. In the southern region of Brazil, DON was detected within the 243–2281 μg/kg range in almost half (47%) of whole wheat grain samples collected during the 2012 crop season [3]. DON levels are also known to vary annually, being significantly affected by weather and farming conditions especially in the years when high prevalence of *Fusarium*, the major DON producing fungus, is reported [4].

DON, also known as vomitoxin, is of significant relevance in agriculture as it causes decreased weight gain, feed refusal, emesis, and diarrhea in livestock, with pigs exhibiting the highest sensitivity. In humans, DON is associated with a large number of symptoms and changes at the cellular and molecular levels [5,6]. Binding to 28S ribosomal RNA causes a cascade of changes that are associated with DON’s immunotoxic and hematotoxic effects [7,8]. Furthermore, some studies show that DON can induce oxidative stress and attribute the observed toxicities to damages resulting from the interaction of reactive oxygen species (ROS) with DNA, lipids, and proteins within the cellular context [9]. Additional mechanisms reported to be involved in DON toxicity include modification to gut microbiota [10,11], disruption of neurohormones [12,13], and with some conflicting results based on model-type, genotoxicity [14,15]. Due to the complex toxicological profile of DON and its sporadic occurrence, it is suggested that the current tolerated limits of DON in human food and animal feed do not provide sufficient protection from the associated risks of DON exposure [4].

In this mini-review we summarize the recent findings related to the mechanism(s) of DON toxicity as well as how structural modifications to DON alter its potency. We also explore the ability of using the freely-available bioinformatics tools and databases in a systematic fashion to elucidate some of the characteristics of DON binding/interacting targets within cells. Furthermore, we shed light on how such knowledge can practically aid in understanding DON toxicity and identifying possible routes of detoxification in nature.

## 2. Cellular Toxicity of DON

Interspecific susceptibility to DON toxicity exists and can be attributed to differences in absorption rates, distribution, metabolism, and excretion. In mono-gastric mammals, DON is efficiently absorbed in the proximal part of the small intestine where at least two ABC transporters are suspected to be involved: P-glycoprotein (ABCB1) and multidrug resistance associated protein 2 (MRP2, ABCC2) [16]. Once across the intestinal epithelial barrier, DON is widely distributed to the majority of tissues with transiently high levels detected in the liver, kidney, spleen, and lungs [17,18].

The toxicological effects of DON emerge mainly from its ability to inhibit DNA and protein synthesis and decrease cell proliferation by binding to the 3' end of 28S ribosomal RNA, an area involved in peptidyl transferase activity, tRNA binding, and ribosomal translocation [19,20]. This interaction occurs in a time-, temperature-, and concentration-dependent manner with one toxin molecule binding per ribosome [21]. It triggers a ribotoxic stress response, and consequently, an up-regulation of apoptotic, immunologic, ER-stress, and oxidative-stress related genes [22]. The ribotoxic stress response begins with signal transduction to double-stranded RNA-activated protein kinase R (PKR) and hematopoietic cell kinase (Hck) [23,24]. This later results in the phosphorylation of mitogen-activated protein kinases (MAPKs) that play key roles in DON toxicity as they regulate cell survival through transcription factor activation and apoptosis [20,23]. Oxygen-containing substituents at the C-15 position as well as the 12,13-epoxide ring found in DON and other trichothecene mycotoxins are thought to be key structural components necessary for ribosomal binding and chain initiation inhibition in eukaryotes [25,26]. For this reason, these positions are often targets of modification in the detoxification of DON.

Multiple studies agree that DON-induced phosphorylation of the MAPKs ERK1/2, JNK, and p38 can cause immune-stimulatory responses via the activation of pro-inflammatory transcription factors and the stabilization of cytokine mRNAs [17,20,27]. For example, elevated splenic expression of TNF-α, IL-1β, IL-6, CXCL-2, CCL-3, and CCL-7 mRNA was documented in mice following oral exposure of 2.5 mg of DON per kg bodyweight [27]. In contrast, at higher doses DON can also evoke immunosuppression by targeting leukocytes for apoptosis and causing *in vivo* destruction of immune tissues such as the thymus and spleen [17,28]. These opposing results suggest that the immunologic response to DON is complex, with possible tissue-specific, dose-dependent, and hormetic-like aspects in play. Islam *et al.* assessed the immune modulation aspects of DON in mice and reported, among other results, increased populations of CD8^+^ cells in the spleen and Peyer’s patches with decreased CD8^+^ populations in intraepithelial and lamina propria lymphocytes of the small intestine. A dichotomous role for DON in down-regulating total IgA levels in serum while up-regulating IgA levels in the duodenum mucosa was also observed [29]. IgA dysregulation that often mirrors human IgA nephropathy is a well-characterized outcome of DON exposure that is thought to be mediated by IL-6, which is in turn mediated by COX-2 [30]. Hirano *et al.* [31] revealed that DON is capable of stabilizing COX-2 mRNA, facilitated by the presence of multiple copies of AUUUA motifs in the 3’-untranslated region of mRNAs [28], and ERK/p38 phosphorylation of TNF-α-converting enzyme (TACE). The latter results in the ectodomain shedding of TNF receptor 1 (TNFRSF1A), thereby inhibiting NF-κB signaling pathway that is involved in regulating transcription of genes such as ICAM-1, cFLIP, and COX-2 [31].

Some of the pathophysiological effects observed following DON ingestion, such as weight loss and diarrhea, may be more clearly attributed to the toxin-induced increase in intestinal barrier permeability. Such a possibility is supported by observed decreases in trans-epithelial electrical resistance (TEER) and increased passage of 4-kDa dextran in DON-treated intestinal epithelial cell lines such as IPEC-1 (porcine) and Caco-2 (human) [20,32]. A reduction in the expression of tight junction proteins claudin-3 and claudin-4 has also been documented for the IPEC-1 cell line when exposed to DON, along with increases in the level of phosphorylated MAPKs ERK 1/2, p38, and JNK proteins [20]. Furthermore, histological investigations show that DON-exposed piglets experience jejunum villus atrophy and that rats experience significant reductions in gastric secretions and subsequent increases in gut pH when gavaged with DON [20,33]. These negative effects on the gastrointestinal tract collectively lead to secondary-pathogen exposure and decreased nutrient absorption due to increased permeability. DON could be further implicated as a factor in chronic gastrointestinal diseases such as Crohn’s disease based on reports that it can modify intestinal microbiota as was observed in weanling pigs as well as human microbiota-associated rats fed naturally-contaminated diets [10,11,34].

Other studies have investigated DON’s effect on neurochemistry and activity for explanatory mechanisms of sickness; however, the results are species-dependent. Interestingly, brain-region specific increases in serotonin (5-HT), a neurotransmitter known to have emetic and food-intake reducing effects, were recorded in rat [12]. In swine intravenously exposed to DON, an initial increase in hypothalamic 5-HT content was also observed, but this route of exposure is not representative of typical exposure through feed and the 5-HT levels diminished with time (8 h post-treatment) [12]. Further research is needed to definitively identify whether DON elicits its effects through the serotonergic pathway. Alternatively, DON-induced activation of pro-opiomelanocortin (POMC) and nesfatin-1 expressing neurons located in the hypothalamus and nucleus tractus solitarius could be correlated to anorexigenic responses [12,35]. POMC neurons typically respond to leptin and insulin, signals of satiety, by releasing α-melanocyte stimulating hormones (α-MSH) that act on melanocortin 3 and 4 receptors to reduce appetite and increase energy expenditure [12,36]. Nesfatin-1 is also linked to the melanocortinergic pathway, but it additionally responds to the anorexinergic hormone cholecystokinin (CCK) [12,37].

CCK and peptide YY (PYY) are both signals of gut satiety that have been shown to increase by 2.5- and 4.1-fold, respectively in response to acute intraperitoneal DON exposure [38]. Using the NPY2 antagonist BIIE0246 and CCK1A receptor antagonist devezapide, it was suggested that PPY may be a critical mediator of DON-induced anorexia, while CCK is not [38]; however, opposing results were found when Wu *et al.* tested CCK1 and CCK2 receptor antagonists SR 27897 and L-365,260. The latter were capable of attenuating exogenous CCK-1/2 and DON-induced anorexia in a dose-dependent fashion, suggesting that CCK may be responsible for the anorexigenic response to DON, rather than PYY [35]. Another experiment, aiming to relate gut satiety hormones with DON exposure, suggested that both PPY and 5-HT play overlapping roles in DON-induced emesis, whereas CCK does not [13]. Clearly, a more focused approach is needed to confirm the importance of these hormones in DON toxicity.

Most recently, Yang *et al*. [14] characterized DON’s ability to induce an oxidative-stress response in human peripheral blood lymphocytes, while Wu *et al.* [39] confirmed such response *in vivo* following dietary exposure to DON-contaminated feed served to piglets. Increases in lipid peroxidation, increases in reactive oxygen species [14,39], and decreases in the cellular levels of glutathione [14] were among the observations that were found. A remedial approach was reported through the addition of glutamic acid to DON-contaminated feed which significantly decreased the observed oxidative-stress caused by the mycotoxin, determined by lower serum malondialdehyde and nitric oxide, and the reduction in microscopic intestinal injury [39]. Further supporting the link between DON and oxidative stress, marked increases in gene expression of the major antioxidant glutathione peroxidase 2 and nitric oxide synthase 2 were found in the intestines of piglets fed naturally-contaminated DON feed (3.5 mg/kg) [40]. In non-tumorigenic intestinal epithelial cell line IEC-6 the pro-oxidant effect of DON was determined to be mediated by NADPH oxidase, calcium homeostasis alteration, the activation of NF-κB and Nrf2 pathways, and by iNOS and nitrotyrosine formation [41]. Nivalenol, a closely related mycotoxin, was found to enhance the pro-oxidant effects on DON as well as pro-apoptotic effects on the IEC-6 cell line [41,42].

To expand on the reported findings that DON can negatively impact oocyte maturation, Zhu *et al.* [43] highlighted the ability of mycotoxins to induce epigenetic changes in mouse oocytes. Following mycotoxin treatment, the methylation status of two major histones, H3 and H4, was altered, with increased levels of H3K9me3 and H4K20me3, and decreased levels of H3K27me3 and H4K20me2 [43]. These epigenetic changes affect chromatin compaction and the cell cycle progression of gametocytes [43]. In other research, DON genotoxicity was assessed using a comet assay, with significant dose-dependent increases in tail length, tail DNA, and tail moment found suggesting that DON potentially damages DNA [14]; however, Takakura *et al*. [15] showed an absence of *in vitro* genotoxicity of DON in bacteria and in human lymphoblastoid TK6 and hepatoma HepaRG cells. As of 1993 the International Agency for Research on Cancer (IARC) has labelled DON as not classifiable as to its carcinogenicity to humans (Category 3) based on inadequate evidence in experimental animals, so more research is required as well as an in-depth reevaluation by IARC in the future [44].

## 3. The Influence of DON Chemical Groups on its Toxicity

**Figure 1:**
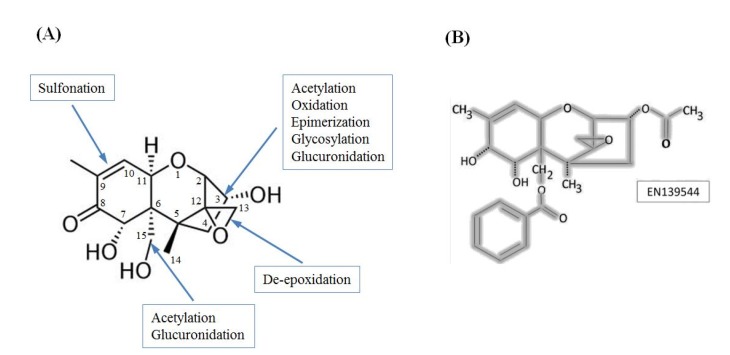
(**A**) Structure of deoxynivalenol with common sites of modification noted (adapted from [52]); (**B**) An example of a pharmacologically relevant derivative of DON, EN139544. EN139544 has a large benzoyloxy group at C-15 and an acetyl group at C-3 in place of the parent hydroxyl groups.

The structure of DON reflects a type B trichothecene mycotoxin with a 12,13-epoxy ring, three hydroxyl groups found at C-3, C-7, and C-15, and a carbonyl function at C-8 (Figure 1a). The unique and conserved configuration of DON chemical groups plays a major role in its elicited toxicity, and changes that alter these groups can affect the overall toxicity of DON derivatives. For example, opening of the 12,13-epoxy ring to form DOM-1 is a known mechanism of microbial detoxification that has been observed in gut microflora of multiple animals including poultry and ruminants [45,46]. DOM-1 was determined to be 55 times less toxic than DON in a BrdU cell toxicity test [47], and at 23 μM it did not affect the viability of porcine peripheral blood mononuclear cells (PBMC), or non-transformed intestinal porcine epithelial cell lines IPEC-1 or IPEC-J2 [48]. In comparison, the estimated IC_50_ of DON for these three cell types was 1.2 ± 0.1, 1.3 ± 0.5, and 3.0 ± 0.8 μM, respectively [48]. This decreased toxicity is most likely explained by a lack of binding to the ribosome due to the loss of the functional epoxide ring.

Another documented pathway in microbial detoxification involves the oxidation of C-3 to produce 3-keto-4-deoxynivalenol (3-keto-DON), which may undergo further conversion to 3-epi-DON [49,50]. The immunosuppressive toxicity of 3-keto-DON was found to be less than one-tenth that of its parent compound, suggesting that the 3-OH group of DON is important for exerting the immunotoxic effects [49]. Since 3-epi-DON has only recently been identified there is presently limited toxicity data pertaining to this derivative. Our animal studies using female B6C3FI mice showed that doses up to 100 mg/kg bodyweight of 3-epi-DON (received as single injection daily for two consecutive weeks) did not affect the mice negatively in the treatment group compared to 2 mg/kg bodyweight DON that significantly lowered the weights of internal organs including liver (*P* < 0.001), kidney (*P* < 0.001) and thymus (*P* < 0.001). Spleen and final bodyweights of mice in the DON treatment group appeared lower than those of the control or 3-epi-DON groups (*P* = 0.095) [51].

In plant and animal metabolic pathways, the hydroxyl groups found at carbons 3 and 15 often become targets of modification. The formation of DON-3-β-D-glucoside (D3G) during plant metabolism acts as a defensive strategy leading to increased mycotoxin tolerance for plants [53,54]. This enzymatic conjugation to glucose, facilitated by plant UDP-glucosyltransferase, reduces the immunotoxicity of DON, with chemokine and interleukin mRNAs levels that are significantly up-regulated by DON remaining unaffected by D3G [27]. D3G is, however, referred to as a masked mycotoxin due to the fact that within the intestinal tract of mammals this derivative is nearly completely hydrolyzed back to the parent compound, DON [53].

In mammalian elimination pathways, ingested DON is typically passed to the urine unchanged, or it can be conjugated within the liver to glucuronides by UDP-glucuronosyltransferases (UGT), forming DON-3-glucuronide (DON-3-Glc) and DON-15-glucuronide (DON-15-Glc) [53]. In humans, the formation of DON-15-Glc is considered the major metabolic pathway for DON with UGT2B4 and UGT2B7 identified as responsible enzyme subtypes [55]. In a kinetic study in pigs, the animal considered the best model for human DON metabolism, results found that 84.8% ± 9.7% of orally administered DON could be detected in the urine within 24 h, with 51.4% ± 6.3% of the original dose excreted unmetabolized, 19.0% ± 6.8% excreted as DON-15-Glc, and 14.5% ± 1.8% as DON-3-Glc [53]. At a concentration of 270 μM, DON-3-Glc was incapable of inhibiting 50% human erythroleukemia cell line K562 viability, compared to an IC_50_ of 1.31 μM for DON, confirming DON-3-GlcA as a detoxification product [56]. This decrease in toxicity may be explained by a lowered ability to cross cell membranes due to increased polarity and size or possibly steric hindrance at the stage of ribosomal binding.

The thia-Michael adduct of DON and methanethiol, known as methylthiodeoxynivalenol (MTD), is another novel plant metabolite that was shown to have reduced cytotoxicity [57]. An *in vitro* translational assay using rabbit reticulocyte lysate revealed MTD to be 11-fold less toxic than DON [57]. Metabolically, this molecule is formed by intramolecular cyclization between C-8 and C-15 of DON, with a glutathione adduct formed in the first step of this multi-stage process [57]. MTD has been identified in both *Fusarium* infected barley and wheat, and is hypothesized to be part of a detoxification pathway that results in reduced DON-ribosome interaction [57].

It has also been demonstrated that EN139544 (Figure 1b), a pharmacologically relevant DON derivative that is efficacious in reducing food intake without causing emesis, is largely incapable of eliciting similar cytokine and chemokine mRNAs up-regulation [27]. The large benzoyloxy group of EN13944 that replaces the C-15 hydroxyl of DON is likely responsible for the decreased toxicity through steric hindrance of ribosomal interactions [27].

Another common modification of DON is the acetylation of the C-3 or C-15 positions to produce the widely-spread grain co-contaminants 3-acetyldeoxynivalenol (3ADON) and 15-acetyldeoxynivalenol (15ADON). Such acetylation was found to alter the cytotoxicity of DON, as well as DON’s capacity to up-regulate cellular cytokines and to activate the MAPK pathway. Decreases in splenic cytokine and chemokine mRNA expressions were observed when mice were orally challenged with 3ADON or 15ADON compared to DON, which implies that these structural modifications lower the inflammatory responses that contribute to the anorexia and weight loss associated with trichothecene toxicity [27]. On the other hand, the intestinal-barrier function was more significantly impaired by 15ADON rather than DON or 3ADON when trans-epithelial electrical resistance (TEER) and the trans-epithelial permeability were investigated using porcine IPEC-1 [20] and human Caco-2 [58] cell lines. Lending to these findings, a decreased expression of claudin-3 and -4 in intestinal epithelial cells was most extensive with 15ADON treatment [20]. Furthermore, 15ADON evoked the most significant increase in MAPK activation [20] and IL-8 secretion [58], reflective of ribotoxic stress and immune stimulation in the intestinal tract. Pinton *et al*. [20] hypothesized that the acetylated position of 15ADON may differentially interact with ribosomes, possibly disrupting the highly conserved sarcin/ricin loop domain, which is influential during the ribotoxic stress response preceding MAPK activation.

**Figure 2:**
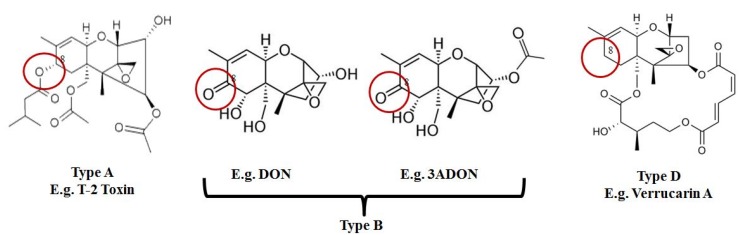
Type B trichothecenes are characterized by a carbonyl group at C-8. In comparison, type A trichothecene mycotoxins may have an –H, –OH, or –OAcyl group at C-8, while type D lack functional groups at C-8 and possess a complex linkage from C-4 to C-15.

Finally, and in regards to the ectodomain shedding of TNF receptor 1, which DON was reported to elicit; 3ADON was found also to possess the same capability; although, this response was associated with much higher 3ADON levels (1 μM for DON in comparison to greater than 10 μM for 3ADON) [31]. It should be noted that neither type A (e.g., T-2 toxin) nor type D (e.g., verrucarin A) trichothecenes that were tested in this study were able to cause similar inhibition, suggesting that the C-8 carbonyl group which differentiates type B trichothecenes (Figure 2), such as DON and 3ADON, from other sub-groups may be important for this specific MAPK pathway [31].

Chemical means of detoxifying DON have also been explored extensively, such as the sulfonation of the 9,10-double bond by sodium metabisulfite (SBS), which yields two highly polar diastereoisomers of 10-DON-sulfonate (DONS) [59]. In a recent investigation to determine the concentration of DONS required to cause 50% inhibition of proliferation, no IC_50_ values could be assigned for four different cell types (porcine PBMC, non-transformed IPEC-1 and IPEC-J2, and human HepG2) with the highest tested concentration being 17 μM [48]. In comparison, IC_50_ values for the parent toxin were 1.18 μM, 1.33 μM, 2.97 μM, and 41 μM, respectively [48]. The toxicity of DONS appears to be significantly lower than that of DON but how these *in vitro* results will transfer to *in vivo* testing is yet to be determined. In one study, SBS-treated DON-contaminated feed failed to induce emesis at the same concentration of DON that caused vomiting in 6/6 tested swine which is considered promising [59]. Still, concerns surrounding DONS include its potential to be converted back to DON under alkaline conditions, and the safety of SBS alone, as SBS is not currently approved as an additive for animal feed [59].

In essence, the scientific literature suggests that most DON derivatives are considered less cytotoxic than the parent compound with the exception of 15ADON, which has been associated with increased MAPK activation, intestinal toxicity, and immunologic toxicity. This raises the question whether the food and feed regulatory bodies (including FDA and CFIA) should consider new regulatory limits for 15ADON (as the case with DON) in grains and animal feed but no action has been taken so far. Due to the fact that mycotoxins rarely occur without co-contaminants, most current investigations have begun to explore the combined toxicological effects of deoxynivalenol and its derivatives combined (acetylated ones for example) with synergistic effects most commonly reported [60].

## 4. Exploring DON Interactions with Cellular Targets

The wide range of changes associated with DON exposure in humans and farm animals cannot be explained solely by the inhibitory effect of DON on cellular protein biosynthesis caused by ribosomal RNA binding of DON. This particular notion is further supported by the diversity of genes and metabolic pathways that are affected following DON exposure as summarized in the earlier sections of this review. Even within the same species/host animals, large variations are observed as different organs, tissues, and cell lines show diverse responses during the course of DON toxicity.

For the aforementioned reasons, a systematic approach is needed to identify the stress-response targets and metabolic pathways involved when reacting to DON exposure [61]. The more we know about the nature of such protein-targets and enzymes, the better our grasp of how cells handle DON-related toxicities will be.

Throughout the literature there are many good examples of proteins/enzymes that recognize and bind DON, with such examples and targets emerging from different branches of life including prokaryotic and eukaryotic domains. Historically, these were originally identified either as part of DON secretory pathways in *Fusarium graminearum* and *F. sporotrichioides*, such as TRI101 (#AB000874.1) [62,63,64,65,66] and TRI7/TRI13 (#AF336365.2/#AF330109.2), or as part of DON detoxification pathways in bacterial isolates and plants such as DON hydroxylase (#AB744215.1) [67] and UDP-glycosyltransferase (#NM_129235.3) [54,68]. While these targets are diverse in their amino acid sequences, structural folds, and cellular functions; all of them share the ability to bind DON within the native cellular environment, making this group of proteins particularly interesting to compare and contrast.

Recently, a number of short polypeptides (83–86 residues) that possess high affinity toward DON were reported (#HM622287.1, #HM622288.1, #HM622289.1) [69]. While the original aim was to identify functional heavy-chain antibodies (HCAbs) that could replace largely variable DON antibodies (extensively used nowadays in ELISA-based competitive assays) with more homogenous and reliable engineered-antibodies, these peptides also offer great insights into the dynamics of DON binding to different biological targets including antibodies, target-proteins, and enzyme active-sites. In the context of this manuscript we will be referring to these peptides as DON-affinity binders.

**Figure 3:**
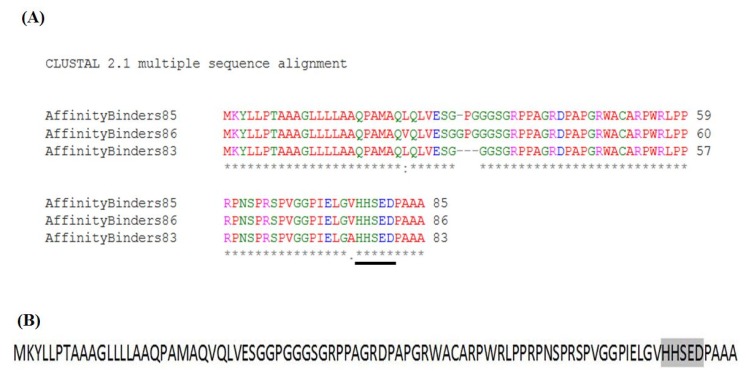
Sequence alignment of DON-Affinity binders [69] reveals highly conserved residues (**A**) that contain a conserved sequence motif _78_*HXXXD*_82_ involved in DON binding/catalysis (**B**, gray highlight).

Trichothecenes are small, amphiphatic, organic molecules that contain aromatic groups [60]. Their hydrophobicity and low molecular-weights make it challenging to reliably identify interacting partners or highlight docking-position on enzymes faces. Two studies here are of particular help to circumvent these obstacles. The study reported by Tu *et al.* [69] highlights the minimum amino acid sequences required to bind DON. In essence, the three reported short polypeptides (83, 85, and 86 amino acids in lengths, respectively) encode for all the essential characteristics needed to recognize DON and cage it within an active pocket. Sequence alignment of these DON-affinity binders shows identical residues and highly-conserved sequence motifs (Figure 3a).

The second important study in elucidating DON binding features is the one which solved the three dimensional structure of *Fusarium graminearum* Tri101 enzyme with DON bound into the enzyme’s active pocket [62]. In this study, Garvey *et al.* highlighted the major residues involved in DON caging and catalysis. Furthermore, the team underlined a major sequence motif that is involved in Tri101 functionality, namely the _156_*HXXXD*_160_ motif. Surprisingly, the same motif can be identified within the DON-affinity binders near their C-termini (Figure 3b with gray highlight).

**Figure 4:**
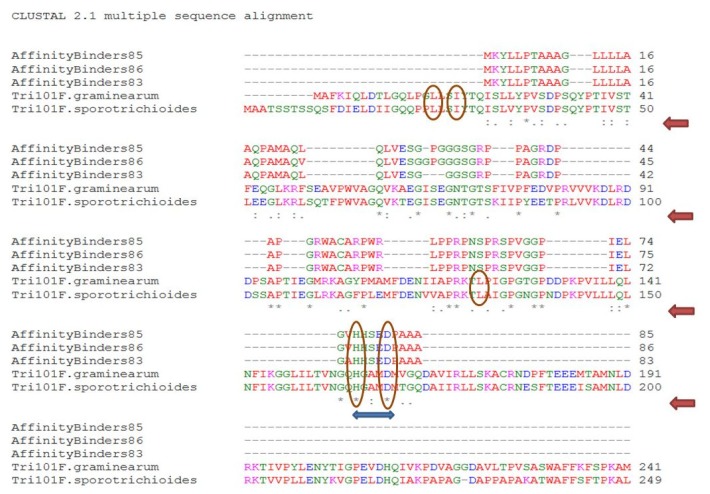
ClustalW multiple sequence alignment of DON-Affinity binders with TRI101 enzymes/isoforms reveals some key amino acids mainly glycine, prolines, and glutamines (noted by under-stars) that are possibly important for DON binding/caging in addition to the highly conserved *HXXXD* motif (double-headed arrow). The catalytic residues of TRI101 reported by [62] are indicated by circles.

In addition, a quick scan for all DON interacting partners listed earlier shows that a similar motif (*HXXXD*/*G*) is exclusively found in these proteins despite belonging to different sub-families or catalyzing different reactions. Furthermore, and in addition to the *HXXXD* motif, sequence alignment of DON-affinity binders with TRI101 isoforms obtained from *Fusarium graminearum* and *F. sporotrichioides* (Figure 4) clearly show that other residues (particularly prolines and glycines) are highly conserved.

While it is still early to elaborate on the importance of such conserved motifs/residues in DON binding and recognition, the structural distinctiveness conferred by the presence of these motives and residues cannot be simply ignored. We hypothesize that by categorizing protein and enzyme-targets based on the presence of such specific sequence-motives, resides, hydrophobicity, side chain charges, and structural folds, the molecular determinants of DON (and its derivatives) bio-toxicity can be better appreciated. In addition, while these structural features might not play any particular roles in the reported catalytic functions, they can be definitely scrutinized for their involvement in scaffolding the enzyme’s active pocket/DON binding sites.

The alignment of nucleotide/amino acid sequences can be highly informative but due to the evolutionary divergence in catalytic folds such investigations should be complemented with three dimensional fold comparisons. When it comes to DON related proteins, the main practical challenges faced here are the scarcity of solved protein structures. In short, very few proteins that bind DON have solved 3D structures with deposited crystals in public protein databases/search servers. While these few reported folds are still informative, they unfortunately all belong to the same catalytic family, acetyltransferases [62].

**Figure 5:**
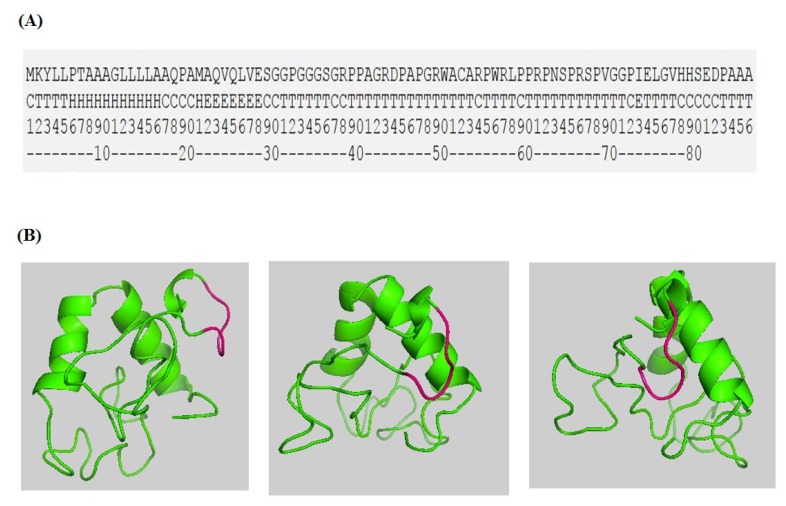
The secondary structure prediction of DON-Affinity binder-86 (originally identified from a phage display library [69]) (**A**) and its tertiary fold (**B**) as predicated by an *ab initio* protein folding approach using Quark server (http://zhanglab.ccmb.med.umich.edu/QUARK/).

For the above stated reasons, other approaches and avenues should be considered. The *ab initio* modeling and structure prediction of polypeptides and proteins is becoming more reliable. A perfect prediction of the three dimensional folds of large proteins is far from accomplishment, yet a reasonable prediction of short peptides is possible. Servers such as Quark (http://zhanglab.ccmb.med.umich.edu/QUARK/) can assemble and predict the 3D folding of short peptides overnight while assigning quality scores (root-mean-square deviation (RMSD)) to the outcome [70]. There are still big uncertainties associated with these fold-predictions but searching and blasting the retrieved outcomes against deposited protein databases can be very insightful. We recently predicted the secondary/tertiary folds of one of DON-affinity binders using Quark server and the outcome model is shown in Figure 5A,B. The predicted _78_*HXXXD*_82_ motif assumes an identical configuration to the _156_*HXXXD*_160_ motif within *F. graminearum* Tri101 enzyme.

The *ab initio* modeling of these short polypeptides opens the door for comparing protein structures in their three dimensional configurations which may reveal biologically interesting similarities that are not detectable by merely comparing nucleotide and amino acid sequences. Servers such as Dali (http://ekhidna.biocenter.helsinki.fi/dali_server/start), a powerful protein 3D structure searching engine, can compare the query-protein coordinates against all those folds deposited within the Protein Data Bank (PDB) and retrieve the matching hits in hours to the overnight range [71].

## 5. Mining Genomes and Proteomes for DON Binding Consensus Motifs

The increasing number of deposited draft- and reference-genomes that span all domains of life (bacteria, archaea, and eukaryotes) makes it very attractive to explore and mine such freely available resources in order to highlight gene clusters or metabolic pathways for their possible involvement in DON bio-toxicity or elimination routes.

As indicated earlier, the ribosomal binding of DON cannot solely explain the diverse symptoms observed and documented in relation to DON bio-toxicity. These observations collectively suggest that other cellular targets are more likely to be involved and that DON binding to these targets could partially explain the wide array of changes connected to DON exposure.

Our laboratory recently reported the isolation of a bacterial strain, designated as *Devosia* sp. 17-2-E-8, which can metabolize DON to epi-3-DON. The conversion process is reproducible under a wide range of conditions (pH, media, and temperature) with 100% efficiency. In our quest to decipher the responsible mechanisms behind this bio-conversion, we pursued whole genome sequencing of *Devosia* sp. 17-2-E-8 in addition to another control strain, namely *D. riboflavin* Strain IFO13584 [72]. Initial comparisons of these two microbial genomes (and annotated proteomes) revealed numerous interesting biosynthetic gene clusters, transcripts, and open-reading frames (ORF). More interestingly, once DON-affinity binders are blasted/searched against these two genomes, a number of novel targets can be highlighted with highly conserved *HXXXD*, proline, and glycine residues. The same approach can be deployed to feasibly search the human genome/proteome for DON-binding targets.

## 6. Conclusions

In conclusion, there are various methods that can be developed for discovering the cellular binding-targets of DON in addition to investigating novel gene clusters involved in its detoxification/elimination. The exploration of such new routes, mechanisms, and DON-binding target/enzymes will lead in our opinion to the revision of DON maximum tolerated levels (and derivatives) in human food and animal feed. It will also lead to the introduction/optimization of agricultural and industrial applications related to DON-detoxification/bio-degradation.

At the same time, understanding the role of DON side groups (and their related modifications) and correlating that with the overall DON toxicological potency has paramount importance from a public health risk perspective. As evident by the accumulating body of scientific literature, different modifications do influence the *in vitro* and *in vivo* toxicity of DON. In theory, evaluating these changes, which can be attributed to different factors such as the derivative’s altered solubility or cellular-targets affinity, can ameliorate the chances in addressing any associated risks.
